# Construction an Implicit Block Multi-Steps Approach for Solving Sixth-Order Fractional Differential Equations

**DOI:** 10.12688/f1000research.172397.1

**Published:** 2025-12-24

**Authors:** Mohammed S. Mechee, Mohammed Mahmood Salih

**Affiliations:** 1 TRDC, university of Kufa, Najaf, Najaf-Iraq, 00964, Iraq; 2 Faculty of Information Technology Ninevah, Ninevah University, Mosel, 00964, Iraq

**Keywords:** Implicit Method; Block Method; Multi-Step Method; ODEs; IVPs; Sixth-Order; FDEs.

## Abstract

**Background:**

In this paper, we focus on deriving an efficient method for solving ordinary differential equations (ODEs) of sixth order, and then, we modify the proposed method for solving fractional differential equations (FDEs).

**Methods:**

The methodology of this paper used the approach of derivation of implicit numerical method. However, the purpose of this study is to derive a direct implicit block and multi-step strategy for solving sixth-order ODEs.

**Results:**

The main novelty of this work lies in the investigation of constructing a novel method for solving FDEs. This paper presents a general block two-point approach, known as GIBM2P, for solving sixth-order initial-value problems (IVPs) using Hermite-interpolating polynomials. Consequently, the FDE problem has been transformed into ODE using the α-fractional-derivative transform, and then, the sixth-order IVP is solved using the proposed GIBM2P method.

**Conclusions:**

In this paper, we offered a method for solving sixth-order FDEs. The proposed method has been shown to be an accurate and efficient method; also, one can obtain the approximate efficient solutions of FDEs problems. The numerical implementations are used to show the high accuracy and efficacy of the proposed GIBM2P approach. However, the GIBM2P method is effective in terms of accuracy, processing time, and the number of function calls.

## 1. Introduction

Differential equations (DEs) are very important in many applications of mathematical models in the applied sciences and engineering, particularly in the physics, electrical engineering, economics, chemistry, and biology. ^1^ However, the mathematical models in the area of applied science, and engineering of actual issues are constructed using DEs techniques. Unfortunately, determining the exact solutions of some kinds of DEs might be somewhat challenging, in spite of numerous efficient common modern or classical analytical and numerical techniques are currently available to the researchers of engineers and scientists. In general, the computation of the analytical solutions of general DEs is sometimes difficult or complicated. Various researchers derived some numerical linear multistep methods for solving IVPs for different orders of ODEs. For the literature reviews (LRs) of this study, we have introduced some studies of numerical methods for solving some classes of ODEs. Firstly, for a review several researchers developed some methods for solving boundary value problems (BVPs) of sixth order ODEs to increase the effectiveness and the accuracy of these approaches ^2– 7^ while Ref. 8 presented a comparison study and a survey of the researches for the generalized RK integrators for solving IVPs of various orders ODEs. Secondly, in likewise, some numerical algorithms of implicit block multistep methods for solving IVPs of ODEs for various orders have been improved by several authors in Refs. 9– 15. Meanwhile, for example, the researchers Refs. 16– 18 derived block backward methods for solving first-order stiff ODEs, as well as Refs. 19– 21 derived one-step, implicit block, block predictor, and 4-step corrector methods for solving a general second-order ODEs. However, the implicit block method and a new strategy of the implicit hybrid block method for solving third-order ODEs are introduced by Refs. 22, 23. In the same manner, ^24^introduced a new special 15-step block method for solving general fourth-order ODEs. In contrast, the general implicit-block method for solving fifth-order ODEs has been derived by Ref. 25 but Turki et al. are constructed a general implicit-block method for solving seventh-order ODEs. ^26^ Moreover, for variable-step block methods, the authors in Refs. 27, 28 have derived a variable-step variable-order block fully implicit method and direct variable-step block multistep method for solving general third-order ODEs. Finally, an explicit multistep block method for solving neutral delay differential equations (DDEs) has been derived by Ref. 29 while Ref. 30 developed three-point diagonally implicit block methods for solving ordinary and fuzzy DEs.

Nowadays, the FDEs and their applications have a significate role in the fields of science and engineering. As a consequence, the researches in finding new approaches for FDEs are increasing. ^31^ As a result, numerous researchers have proposed new definitions for fractional derivatives. For instance, the α-fractional-derivative or integral of real functions was defined in a novel way by Mechee et al., which is used in this article. ^32^ Also, Khalil et al. and Z. Zheng et al. ^33, 34^offered more new definitions of the fractional derivative. This article utilizes the fundamental functions of a Hermite-interpolating polynomial to propose a general implicit block two-point multistep approach, called GIBM2P, for solving the IVPs of sixth-order ODEs. Furthermore, this new technique has been used for solving sixth-order FDEs by converting FDE to ODE using α-fractional derivative.

## 2. Preliminary and background

Several concepts and definitions have been introduced in this section.

### 2.1 Quasi-Linear Sixth-Order Ordinary Differential Equations

The general formula for the sixth-order ODE’s initial value problem can be expressed as follows ^7^:

$$\Psi \left(\zeta ,u\left(\zeta \right),u^{\prime }\left(\zeta \right),u^{\prime \prime }\left(\zeta \right),u^{\prime \prime \prime }\left(\zeta \right),u^{\left(4\right)}\left(\zeta \right),u^{\left(5\right)}\left(\zeta \right),u^{\left(6\right)}\left(\zeta \right)\right)=0,\;a\leq \zeta \leq b$$ (1)

with the initial-conditions (ICs),

$$u^{\left(j\right)}\left(a\right)=\zeta^{j},j=0,1,2,\ldots ,5.$$ (2)

while the initial-values problem of the special quasi-linear ODEs of sixth-order can be given as follows:

$$u^{\left(6\right)}(\zeta )=\phi \left(\zeta ,u\left(\zeta \right),u^{\prime }\left(\zeta \right),u^{\prime \prime }\left(\zeta \right),u^{\prime \prime \prime }\left(\zeta \right),u^{\left(4\right)}\left(\zeta \right),u^{\left(5\right)}\left(\zeta \right)\right),\;a\leq \zeta \leq b$$ (3)

with the ICs in Equation (2).

Definition 2.1: General Class of Sixth-Order FDEs

Below is the definition of the general class of sixth-order FDEs.

$$\phi \left(\zeta ,u\left(\zeta \right),u^{\left(j\right)}\left(\zeta \right),u^{\left(\mathrm{j\alpha}\right)}\left(\zeta \right)\right)=0,\;j=1,2,\ldots ,6;0<\alpha <1,\;a\leq \zeta \leq b,$$ (4)

with the ICs,

$$u^{\left(j\alpha \right)}\left(a\right)=\zeta^{j},\;j=0,1,2,\ldots ,5.$$ (5)

Definition 2.2: Special Cass of Quasi-Linear FDEs of Sixth-Order

The following formula illustrates the class of sixth-order FDEs:

$$u^{\left(6\alpha \right)}\left(\zeta \right)=f\left(\zeta ,u\left(\zeta \right),u^{\left(j\right)}\left(\zeta \right),u^{\left(\mathrm{j\alpha}\right)}\left(\zeta \right)\right),\;a\leq \zeta \leq b;\;0<\alpha <1;\;j=1,2,\ldots ,5.$$ (6)

with the ICs in Equation (5).

### 2.1 Fractional derivatives

In this subsection, we introduced the α-fractional derivative of a function

Definition 2.3: α-Fractional-derivative ^15^

Mechee et al. ^32^ introduced a novel α-fractional-derivative definition for the real function ϕ(ζ):[a,∞)→R by following:

$$T_{\alpha }\left(\phi \left(\zeta \right)\right)=\phi^{\left(\alpha \right)}\left(\zeta \right)=\lim_{\epsilon \to 0}\frac{\phi \left(\zeta +\epsilon \zeta^{1-\alpha }\right)-\phi \left(\zeta -\epsilon \zeta^{1-\alpha }\right)}{2\epsilon },$$ (7)

for α∈(0,1).

From the α-fractional derivative in Definition 2.3, we get the following property of α-fractional derivative of the function ϕ(ζ).

Property 2.1 ^15^Consider ϕ(ζ):[a,b)→R be a real function.

Then, the α-fractional-derivative of the function ϕ(ζ) defined in the Equation (7) has the following property:

$$T_{\alpha }\left(\phi \left(\zeta \right)\right)=\zeta^{1-\alpha }\frac{\mathrm{d\phi}\left(\zeta \right)\;}{\mathrm{d\zeta}}.$$ (8)

## 3. Proposed method

We introduce the analysis of GIBM2P method which approximate the solution of IVP using Hermit polynomials in this section.

### 3.1 Analysis of the proposed numerical GIBM2P method

The form of a Hermit polynomial is defined as follows:

$$P_{2}\left(\xi \right)=\sum_{i=0}^{m}\sum_{k=0}^{m_{i}-1}\phi_{i}^{\left(k\right)}L_{\mathrm{ik}}\left(\xi \right).$$

Consider $h=\frac{b-a}{m}$ and $\xi_{i}=a+\mathrm{ih}$ and $f_{i}=f\left(\xi_{i},y_{i},y_{i}^{\prime },y_{i}^{\prime \prime }\right),$ for i=0,1,…,m where *m* = number of partitions in domain interval which is equal to the number of Lagrange’s polynomials and $m_{i}-1$ is the degree of Lagrange polynomial. In this case, $L_{\mathrm{ik}}\left(\xi \right)\;$ is the generalized Lagrange polynomial for *m* = 0,1,2, …, $m_{i}$ , with a step-size equal to 2 *h*, and $y_{m+1}$ and $y_{m+2}$ are the approximation generates the approximated solutions at two-points $\xi_{m+1}\;$ and $\xi_{m+2}$ resp. In the block interval $\left[\xi_{m},\xi_{m+2}\right],$ we have, $\xi_{m}$ is the starting-point while $\xi_{m+2}$ is the end-point. However, the initial value for all next iterations should be the approximated solution by $y_{m+2}\;$ at the end-point $\xi_{m+2}$

$$P_{2}\left(\xi \right)=f_{0}L_{00}\left(\xi \right)+f_{1}L_{10}\left(\xi \right)+f_{2}L_{20}\left(\xi \right)+g_{0}L_{01}\left(\xi \right)+g_{1}L_{11}\left(\xi \right)+g_{2}L_{21}\left(\xi \right),$$ (9)

3.1.1 **Lagrange polynomials**

Lagrange polynomials are defined as follows in this article:

$$L_{00}\left(\lambda \right)={\left(\lambda -\lambda_{n})(1-\chi_{1}\right)\left(\frac{\left(\lambda_{n+2}-\lambda \right)\;\left(\lambda_{n+1}-\lambda \right)}{\left(\lambda_{n}-\lambda_{n+2}\right)\;\left(\lambda_{n}-\lambda_{n+1}\right)}\right)}^{2},$$ (10)

$$L_{10}\left(\lambda \right)={\left(\lambda -\lambda_{n+1})(1-\chi_{2}\right)\left(\frac{\left(\lambda -\lambda_{n}\right)\left(\lambda_{n+2}-\lambda \right)}{(\lambda_{n+1}-\lambda_{n})\left(\lambda_{n+1}-\lambda_{n+2}\right)}\right)}^{2},$$ (11)

$$L_{20}\left(\lambda \right)={\left(\lambda -\lambda_{n+1}\right)\left(1-\chi_{3}\right)\left(\frac{\left(\lambda -\lambda_{n}\right)\left(\lambda_{n+1}-\lambda \right)}{(\lambda_{n+2}-\lambda_{n})\left(\lambda_{n+2}-\lambda_{n+1}\right)}\right)}^{2},$$ (12)

$$L_{01}\left(\lambda \right)=\left(\lambda -\lambda_{n}\right){\left(\frac{\lambda_{n+1}-\lambda }{\lambda_{n}-\lambda_{n+1}}\right)}^{2}\;{\left(\frac{\lambda_{n+2}-\lambda }{\lambda_{n}-\lambda_{n+2}}\right)}^{2},$$ (13)

$$L_{11}\left(\lambda \right)=\left(\lambda -\lambda_{n+1}\right){\left(\frac{\left(\lambda -\lambda_{n}\right)\left(\lambda_{n+2}-\lambda \right)}{\left(\lambda_{n+1}-\lambda_{n}\right)\left(\lambda_{n+1}-\lambda_{n+2}\right)}\right)}^{2},$$ (14)

$$L_{21}\left(\lambda \right)=\left(\lambda -\lambda_{n+2}\right){\left(\frac{\left(\lambda_{n}-\lambda \right)\left(\lambda_{n+1}-\lambda \right)}{\left(\lambda_{n+2}-\lambda_{n}\right)\left(\lambda_{n+2}-\lambda_{n+1}\right)}\right)}^{2},$$ (15)

where,

$$\chi_{1}=\frac{2}{\lambda_{n}-\lambda_{n+2}}+\frac{2}{\lambda_{n}-\lambda_{n+1}},\chi_{2}=\frac{2}{\lambda_{n+1}-\lambda_{n+2}}+\frac{2}{\lambda_{n+1}-\lambda_{n}},\chi_{3}=\frac{2}{\lambda_{n+2}-\lambda_{n+1}}+\frac{2}{\lambda_{n+2}-\lambda_{n+1}},$$

The expressions for Lagrange polynomials in the independent variable s are as follows, assuming that $s=\frac{\lambda -\lambda_{n+2}}{h}$ and,

$$L_{00}\left(\lambda \right)=\frac{1}{4}{\left(\;\beta_{1}\;\beta_{2}\right)}^{2}+3\;\beta_{3},$$ (16)

$$L_{10}\left(\lambda \right)={\left(\lambda \;\beta_{3}\right)}^{2},$$ (17)

$$L_{20}\left(\lambda \right)=\frac{1}{4}\;\;\beta_{3}{\left(\;\beta_{2}\right)}^{2}\;\beta_{4}$$ (18)

$$L_{01}\left(\lambda \right)=\frac{h}{4}\;\beta_{3}{\left(\;\beta_{1}\;\beta_{2})\right)}^{2},$$ (19)

$$L_{11}\left(\lambda \right)=h{\left(\lambda \;\beta_{3}\right)}^{2}\;\beta_{2},$$ (20)

and

$$L_{21}\left(\lambda \right)=\frac{h}{4}{\left(\beta_{3}\;\beta_{2}\right)}^{2}.$$ (21)

where $\beta_{1}=\lambda ,\beta_{2}=\lambda +1,\beta_{3}=\lambda +2,\beta_{4}=1-3\lambda$ .

**3.1.2 Construction of the numerical GIBM2P approach**

The approximated solution $u_{n}\left(\zeta \right)\;$ of Equation (3) at the initial point $\zeta_{n}$ can be obtained by multiple-integrating Equation (3) up to five times with respect to the independent variable ξ along the range $\left[\zeta_{n};\zeta_{n+1}\right],$ then, compute these integrations to obtain the formulas in Equation (22).

$$u_{n+1}^{\left(6-j\right)}=\sum_{k=6-j}^{5}\frac{h^{6-j-k}}{\left(6-j-k\right)!}v_{n}^{\left(k\right)}+\int_{t_{n}}^{t_{n+1}}\psi (\lambda ,u\left(\lambda \right)d\lambda ,\;j=1,2,\ldots ,6$$ (22)

By assuming that $t_{n+2}=t-\mathrm{hs}$ , the coordinate *t* is changed to *s*.

The following six formulas were created by integrating the Equation (22)

$$u_{n+1}^{\left(j\right)}={∆}_{6-j}+\sum_{k=1}^{3}a_{\mathrm{jk}}f_{n+k}+a_{\mathrm{jk}+3}g_{n+k},\;\text{for}\;j=5,4,3,2,1,0$$

where,

$$A=\left(a_{\mathrm{ij}}\right)=\left(\begin{array}{ll} \begin{array}{ccc} \frac{101}{240} & \frac{8}{15} & \frac{11}{240}\\ \frac{13}{42} & \frac{1}{6} & \frac{1}{42}\\ \frac{817}{6720} & \frac{4}{105} & \frac{47}{6720} \end{array} & \begin{array}{ccc} \frac{13h}{240} & -\frac{h}{6} & -\frac{h}{80}\\ \frac{59}{1680} & \frac{8}{105} & \frac{11}{1680}\\ \frac{83}{6720} & \frac{1}{48} & \frac{13}{6720} \end{array}\\ \begin{array}{ccc} \frac{67}{2016} & \frac{1}{144} & \frac{1}{672}\\ \frac{1690}{241920} & \frac{1}{945} & \frac{61}{241920}\\ \frac{577}{475200} & \frac{1}{7200} & \frac{17}{475200} \end{array} & \begin{array}{ccc} \frac{37}{12069} & \frac{4}{945} & \frac{5}{12096}\\ \frac{143}{241920} & \frac{1}{1440} & \frac{17}{241920}\\ \frac{179}{1900800} & \frac{1}{10395} & 19008 \end{array} \end{array}\right)$$ (23)

and,

$$\;{∆}_{j}=\sum_{k=6-j}^{5}\frac{h^{6-j-k}}{\left(6-j-k\right)!}v_{n}^{\left(k\right)},\;\text{for}\;j=1,2,\ldots ,6.$$

## 4. Main results

### 4.1 Analysis of proposed approach for solving of FDEs sixth-order

In this subsection, the analysis of the proposed method for solving FDEs of sixth-order is introduced and studied. In Equation (7), Mechee et al. presented a new definition of the α-fractional-derivative for the real function f(τ):[a,∞)→R. From the α-fractional- derivative in Definition 2.3, we get the property of α-fractional-derivative of the function ϕ(ζ) in Equation (8). Consequently, by deriving the two sides of Equations (8) five times to get the sixth α-fractional-derivative of the function f(τ) . Thus, the α-fractional-derivative of sixth-order for the real function f(τ) in the domain $I_{\alpha }$ , denoted by $T_{6\alpha }$ , has the following definition:

$$T_{6\alpha }\left(f\left(\zeta \right)\right)=\zeta^{1-6\alpha }\;(\zeta^{5}f^{\left(6\right)}\left(\zeta \right)+15\left(1-\alpha \right)\zeta^{4}f^{\left(5\right)}\left(\zeta \right)+5\left(1-\alpha \right)\left(13-7\alpha \right)\zeta^{3}f^{\left(4\right)}\left(\zeta \right)+\left(1-\alpha \right)\left(15\left(5-7\alpha \right)+2\left(1-2\alpha \right)\left(7-11\alpha \right)\right)\zeta^{2}f^{\left(3\right)}\left(\zeta \right)+2\left(1-\alpha \right)\left(1-2\alpha \right)\left(7-11\alpha \right)\left(2-5\alpha \right)\zeta f^{\prime \prime }\left(\zeta \right)+\left(1-\alpha \right)\left(1-2\alpha \right)\left(1-3\alpha \right)\left(1-4\alpha \right)\left(1-5\alpha \right)f^{\prime }\left(\zeta \right)),$$

which is simplified to the following

$$T_{6\alpha }\left(f\left(\zeta \right)\right)=\zeta^{1-6\alpha }\left(\zeta^{5}f^{\left(6\right)}\left(\zeta \right)+15\left(1-\alpha \right)\zeta^{4}f^{\left(5\right)}\left(\zeta \right)+\left(1-\alpha \right)\left(5\left(13-7\alpha \right)\zeta^{3}f^{\left(4\right)}\left(\zeta \right)+\left(15\left(5-7\alpha \right)+2\left(1-2\alpha \right)\left(7-11\alpha \right)\right)\zeta^{2}f^{\left(3\right)}\left(\zeta \right)+2\left(1-2\alpha \right)\left(7-11\alpha \right)\left(2-5\alpha \right)\zeta f^{\prime \prime }\left(\zeta \right)+\left(1-2\alpha \right)\left(1-3\alpha \right)\left(1-4\alpha \right)\left(1-5\alpha \right)f^{\prime }\left(\zeta \right)\right)\right).$$ (24)

To prove Equation (24), by using α -fractional-derivative five times for two sides of Equation (8) to obtain the formula in Equation (24)

### 4.2 Proposed analytical method

In this subsection, using the α-fractional derivative property from Equation (24), the FDE in Equation (6) with ICs in Equation (5) has been transformed into an ODE in Equation (3) with ICs in Equation (2) and then, using GIBM2P approach to obtain the numerical solution of the IVP.

**4.2.1 The proposed method’s algorithm**

The solution of FDE in Equation (6) with the ICs in Equation (5) has been introduced the proposed method using the following algorithm:

4.2.1.1 **The Proposed Algorithm for Solving Sixth-Order FDEs**

Step I: Convert the FDE in Equation (6) with ICs in Equation (5) into an ODE in Equation (3) with ICs in Equation (2).

Step II: Utilizing an appropriate analytical technique, or the proposed GIBM2P approach, solve the ODE in Equation (3) with the ICs in Equation (2).

Step III: The analytical or numerical solution for the IVP in step II is consequently exactly the same solution of given FDE problem in Equation (6) with the ICs in Equation (5).

## 5. Implementation

**Figure 1.**
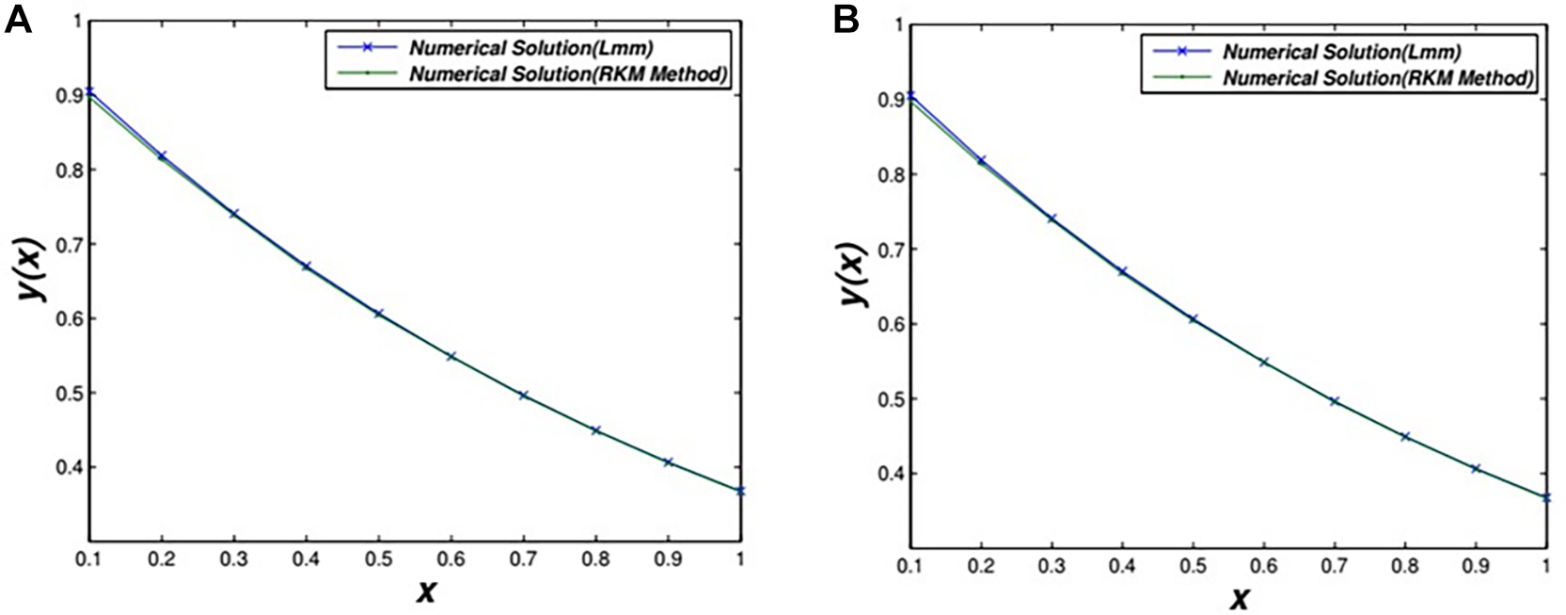
A comparison of the numerical solutions for (A) Example 5.1 and (B) Example 5.2 using the proposed GIBM2P method with RKM method.

The performance of the numerical GIBM2P approaches was evaluated in this section since we transformed the FDE in Equation (6) to a sixth-order ODE and then solved it using proposed strategy specified in Equation (23) compared with Taylor method. The comparison of numerical solutions of proposed GIBM2P method against Taylor method is shown in Figure 1. The following is how the notations were used:

• GIBM2P: A two-point general implicit block approach is proposed.

Example 5.1: Linear FDE ^31^

$$\eta^{\left(6\alpha \right)}\left(\zeta \right)=\eta \left(\zeta \right)+\eta^{\prime }\left(\zeta \right)-3\eta^{\prime \prime }\left(\zeta \right)-11\eta^{\prime \prime \prime }\left(\zeta \right);0<\alpha <1;0<\zeta \leq 1$$.

With the ICs: $\eta^{\left(\kappa \alpha \right)}\left(0\right)={\left(-1\right)}^{\kappa }\;$ for κ=0,1,2,3,4,5.

Using the formula in Equation (24), we get the following ODE

$$T_{6\alpha }\left(f\left(\zeta \right)\right)=\zeta^{\frac{-39}{9}}\left(\zeta^{5}f^{\left(6\right)}\left(\zeta \right)+\frac{15}{9}\zeta^{4}f^{\left(5\right)}\left(\zeta \right)\;\frac{305}{81}\zeta^{3}f^{\left(4\right)}\left(\zeta \right)-\frac{1135}{729}\zeta^{2}f^{\left(3\right)}\left(\zeta \right)-\frac{7700}{6561}\zeta f^{\prime \prime }\left(\zeta \right)+\frac{74865}{59049}f^{\prime }\left(\zeta \right)\right).$$

Hence,

$$T_{6\alpha }\left(f\left(\zeta \right)\right)=2\zeta^{\frac{2}{3}}f^{\left(6\right)}\left(\zeta \right)+\zeta^{\frac{-39}{9}}\left(\frac{15}{9}\zeta^{4}f^{\left(5\right)}\left(\zeta \right)\;\frac{305}{81}\zeta^{3}f^{\left(4\right)}\left(\zeta \right)-\frac{1135}{729}\zeta^{2}f^{\left(3\right)}\left(\zeta \right)-\frac{7700}{6561}\zeta f^{\prime \prime }\left(\zeta \right)+\frac{74865}{59049}f^{\prime }\left(\zeta \right)\right),$$

With the ICs: $\eta^{\left(k\right)}\left(0\right)={\left(-1\right)}^{\kappa }\;$ for κ=0,1,2,3,4,5.

Example 5.2: Non-Linear FDE ^31^

$$\eta^{\left(6\alpha \right)}\left(\zeta \right)=\eta \left(\zeta \right)\;\eta^{\prime }\left(\zeta \right)-3\eta^{\left(5\right)}\left(\zeta \right)-11\eta^{\prime \prime \prime }\left(\zeta \right);\;0<\alpha <1;0<\zeta \leq 1$$.

With the ICs: $\eta^{\left(\kappa \alpha \right)}\left(0\right)={\left(-1\right)}^{\kappa }\;$ for κ=0,1,2,3,4,5.

Using the formula in Equation (24), we get the following ODE

$$\zeta^{5}\eta^{\left(6\right)}\left(\zeta \right)+15\left(1-\alpha \right)\zeta^{4}\eta^{\left(5\right)}\left(\zeta \right)+5\left(1-\alpha \right)\left(13-7\alpha \right)\zeta^{3}\eta^{\left(4\right)}\left(\zeta \right)+\left(1-\alpha \right)\left((15\left(5-7\alpha \right)+2\left(1-2\alpha \right)\left(7-11\alpha \right))\right)\zeta^{2}\eta^{\left(3\right)}\left(\zeta \right)+2\left(1-\alpha \right)\left(1-2\alpha \right)\left(7-11\alpha \right)\left(2-5\alpha \right)\zeta \eta^{\prime \prime }\left(\zeta \right)+\left(1-\alpha \right)\left(1-2\alpha \right)\left(1-3\alpha \right)\left(1-4\alpha \right)\left(1-5\alpha \right)\eta^{\prime }\left(\zeta \right)=\zeta^{6\alpha -1}\;\left(\eta \left(\zeta \right)\;\eta^{\prime }\left(\zeta \right)-3\eta^{\left(5\right)}\left(\zeta \right)-11\eta^{\prime \prime \prime }\left(\zeta \right)\right)$$

with the ICs: $\eta^{\left(k\right)}\left(0\right)={\left(-1\right)}^{\kappa }\;$ for κ=0,1,2,3,4,5.

## 6. Discussion and Conclusion

The two objectives of this article are to convert the FDE to an ODE and to derive a direct-implicit block numerical method for solving a class of ODE. The current paper developed and analyzed the method of converting a sixth-order quasi-linear FDE to an ODE and then solving this ordinary differential equation using a novel direct implicit block technique with two points, which is named GIBM2P. Accordingly, we can conclude that the proposed method GIBM2P is powerful and efficient. From the discussion of the results of this paper of the two examples of implementation, we may conclude that the proposed approach performs efficiently and accurately.

## Data Availability

No data associated with this article.
