# Supplementary material for: Construction an Implicit Block Multi-Steps Approach for Solving Sixth-Order Fractional Differential Equations
Source: F1000Res. 2026 Jun 8;14:1444. Originally published 2025 Dec 24. [Version 2] doi: 10.12688/f1000research.172397.2 (PMC13329363; doi:10.12688/f1000research.172397.2)
Supplement: Peer review history

# Reviewer report: Reviewer response for version 2

**DOI:** 10.5256/f1000research.198675.r493126

**Published:** 2026-07-02

**Authors:** souhila sabit

**Affiliations:** 1 Universite Ibn Khaldoun Tiaret, Tiaret, Tiaret Province, Algeria

The manuscript talks about a method that uses something called Generalized Implicit Block Method (GIBM2P) along with Hermite–Lagrange interpolation to solve sixth-order fractional differential equations. This topic is really important. The approach they are suggesting could be very useful for dealing with higher-order fractional problems. The manuscript is easy to follow. The examples they give show that this method can give us pretty accurate answers.

 There are some big issues that need to be fixed before we can even think about indexing this manuscript. For example, some of the derivations are not complete and it looks like there are some mistakes in the formulas. Also, the authors do not give us details, about how they actually did the calculations so we cannot repeat their work to check if it is correct. These are problems that need to be addressed. The Generalized Implicit Block Method (GIBM2P) and Hermite–Lagrange interpolation are ideas, but we need to make sure they are used correctly and that the math is right.

**Major Comments**

 1. **Incomplete derivation of Hermite–Lagrange basis functions**

 The derivation of Hermite basis functions in Equations 16 to 21 needs explanation.

 The mentioned expressions seem to be missing some factors.

 This might be a mistake. The authors should double-check Equations 16 to 18.

 2. **Missing derivation of the coefficient matrix**

 The manuscript just shows the coefficient matrix A without explaining how it is calculated.

 The coefficients should come from integrating the Hermite basis functions or something similar.

 Because this explanation is not provided, we cannot verify the values, in the matrix.

 The Hermite basis functions are crucial here.

 3. **Integral representation**

 The equation (22) needs explanation.

 When doing integrations the integral part should usually have a special factor.

 The explanation should clearly show how this part is changed into the numbers in matrix (A).

 4. **Numerical implementation**

 The way the GIBM2P algorithm is put into practice is not clearly described.

 The document does not explain

 * How the list of function results is made,

 * How the hidden nonlinear problem is fixed,

 * Which guess is used

 * When to stop,

 * Or the full step-, by-step computational method.

 These points are vital for others to repeat the work.

 5. **Numerical examples**

 Examples 5.1 and 5.2 only give the numerical answers.

 In Example 5.1 the value of the fractional-order parameter α is not clearly given before the calculations are done. The equation that was changed shows that a certain value of the fractional-order parameter α was used. This choice should be clearly stated and explained why it was made. The value of the fractional-order parameter α is important. It should be clear what it is.

 The examples given do not look at how the fractional-order parameter α affects things.

 The authors really should show numerical results for different values of α.

 They should also talk about how α impacts the accuracy and convergence of their proposed method.

 The document should have least one full numerical step showing

 * How the number list is made,

 * How matrix (A) is used,

 *. How the next solution part is calculated.

 Without these details it is hard to get the results.

**Minor Comments**

 * The thing that is written as (v_n) is used without being explained. We should add an explanation of what this vector means.

 * We need to double check the numbers in the block formulas. For example, the part that says (a_{j+3,8}) does not seem to match the size of the coefficient matrix. It is probably a mistake.

 * The way things are written changes suddenly in some places, which makes it hard to understand.

 * If we added a step-by-step guide that explains the whole GIBM2P algorithm it would make the paper a lot better.

**Strengths**

 * This paper is, about a problem that is interesting and important.

 * The new method seems to give good approximations.

 * When we compare the numbers, we get with the answers they match very well.

 * The way the paper is organized is okay.

 The GIBM2P algorithm and the manuscript are important to understand the GIBM2P algorithm is the topic of the manuscript.

**The conclusion**

 The conclusion is that the results show the suggested method works for the test problems... We only partly agree because some important details are left out.

 * The authors do not fully explain how they build the coefficient matrix.

 * They also do not clearly describe the steps in their calculations.

 * The complete GIBM2P algorithm is not provided, making it hard to check and repeat their results.

**Recommendation**

 The authors need to provide explanations of the Hermite basis functions and the coefficient matrix.

 They should also make the integral formulation clearer. Correct any notation mistakes.

 * Additionally, they must include all the details of their algorithm.

 This will ensure that their method can be fully repeated and verified.

Is the work clearly and accurately presented and does it cite the current literature?

Yes

If applicable, is the statistical analysis and its interpretation appropriate?

Partly

Are all the source data underlying the results available to ensure full reproducibility?

No

Is the study design appropriate and is the work technically sound?

Partly

Are the conclusions drawn adequately supported by the results?

Partly

Are sufficient details of methods and analysis provided to allow replication by others?

Partly

Reviewer Expertise:

Numerical methods

I confirm that I have read this submission and believe that I have an appropriate level of expertise to confirm that it is of an acceptable scientific standard, however I have significant reservations, as outlined above.

# Reviewer report: Reviewer response for version 1

**DOI:** 10.5256/f1000research.190118.r460000

**Published:** 2026-03-02

**Authors:** Peter Olatunji

**Affiliations:** 1 Adekunle Ajasin University, Akungba, Ondo, Nigeria

The article “Construction an Implicit Block Multi-Steps Approach for Solving Sixth-Order Fractional Differential Equations” presents a general implicit block two-point method (GIBM2P) based on Hermite-interpolating polynomials for solving sixth-order ordinary differential equations and extends it to fractional differential equations via an \alpha-fractional-derivative transform. Overall, the manuscript is well structured and generally mathematically clear and, while it cites relevant foundational and recent literature on block methods and fractional derivatives, the linkage to very recent advances is moderate rather than comprehensive; the study design, derivation of method, transforming FDEs to ODEs, and implementing with linear and nonlinear examples is appropriate and technically coherent, though stability and convergence analysis are not rigorously developed; sufficient algorithmic and formula details are provided to allow replication in principle, and the conclusions regarding efficiency and accuracy are broadly supported by the illustrative examples.

Is the work clearly and accurately presented and does it cite the current literature?

Yes

If applicable, is the statistical analysis and its interpretation appropriate?

Not applicable

Are all the source data underlying the results available to ensure full reproducibility?

No source data required

Is the study design appropriate and is the work technically sound?

Yes

Are the conclusions drawn adequately supported by the results?

Yes

Are sufficient details of methods and analysis provided to allow replication by others?

Yes

Reviewer Expertise:

Numerical Analysis, Scientific Computing, Mathematical Biology and Data Science

I confirm that I have read this submission and believe that I have an appropriate level of expertise to confirm that it is of an acceptable scientific standard.

# Response

**Published:** 2026-03-11

**Authors:** Mohammed Salih

**Affiliations:** Software, Ninevah University, Mosul, Nineveh Governorate, Iraq

Thank you for reviewing.

# Reviewer report: Reviewer response for version 1

**DOI:** 10.5256/f1000research.190118.r448329

**Published:** 2026-02-05

**Authors:** Yahya Qaid Hasan

**Affiliations:** 1 Thamar University, Dhamar, Yemen

**Manuscript Title: Construction an Implicit Block Multi-Steps Approach for Solving Sixth-Order Fractional Differential Equations**

**Recommendation: Minor Revision**

**General Evaluation**

 The manuscript presents an interesting and mathematically sound approach (GIBM2P) for solving sixth-order fractional differential equations (FDEs). The logic of transforming FDEs into sixth-order ordinary differential equations (ODEs) using the \alpha-fractional-derivative transform is well-reasoned and efficient. The numerical results provided for both linear and non-linear examples indicate that the proposed method is highly accurate and competitive with established methods like the RKM and Taylor methods.

**Strengths of the Manuscript**

  * Novelty: The construction of a specific two-point block method (GIBM2P) for sixth-order IVPs fills a gap in current numerical literature.

  * Methodology: The use of Hermite-interpolating polynomials provides a robust foundation for the implicit multi-step strategy.

  * Clarity of Implementation: The algorithm for converting FDEs to ODEs is clearly outlined in three logical steps.

**Required Revisions for the Authors**

 To ensure the manuscript meets the highest standards for final indexing, the authors must address the following points:

 1. Title and Linguistic Accuracy

  * Correction: The title contains a grammatical error: "Construction an Implicit...". It should be corrected to "Construction of an Implicit..." or "Constructing an Implicit...".

  * Typographical Consistency: Correct the spelling of "Hermite" (currently written as "Hermit" in several instances on page 4).

  * Terminology: In Section 3.9, change "significate" to "significant".

 2. Mathematical Presentation and Formatting

  * Matrix A (Page 6): The elements within Matrix A (Equation 23) are poorly formatted and overlap, making them difficult to verify. The authors must re-format this matrix using a proper LaTeX environment to ensure all fractions and values are clearly separated.

  * Notation Clarity: In Definition 2.2, please clarify the notation u^{(\overline{j})}(\zeta) to ensure it is not confused with standard derivatives.

 3. Theoretical and Numerical Enhancements

  * Stability and Convergence: Although the numerical results are promising, a brief theoretical discussion or proof regarding the stability and convergence of the GIBM2P method would significantly add value to the paper.

  * Comparative Data: Instead of relying solely on Figure 1, please provide a table showing the Absolute Errors or CPU Time for the examples provided to substantiate the claims of "high accuracy and efficacy".

 4. References

  * Ensure that reference citations in the text match the final reference list. For example, verify if Reference 8 in the text accurately points to the survey study mentioned.

**Conclusion:**

 Upon addressing these minor but essential revisions, the manuscript will be a valuable contribution to the field of numerical analysis. I look forward to reviewing the revised version.

Is the work clearly and accurately presented and does it cite the current literature?

Partly

If applicable, is the statistical analysis and its interpretation appropriate?

Partly

Are all the source data underlying the results available to ensure full reproducibility?

Yes

Is the study design appropriate and is the work technically sound?

Yes

Are the conclusions drawn adequately supported by the results?

Yes

Are sufficient details of methods and analysis provided to allow replication by others?

Yes

Reviewer Expertise:

Adomian decomposition method , Differential  Equations,

I confirm that I have read this submission and believe that I have an appropriate level of expertise to confirm that it is of an acceptable scientific standard, however I have significant reservations, as outlined above.

# Response

**Published:** 2026-02-25

**Authors:** Mohammed Salih

**Affiliations:** Software, Ninevah University, Mosul, Nineveh Governorate, Iraq

Thank for your reviewing
